# Evaluation of Bayesian classifiers in asthma exacerbation prediction after medication discontinuation

**DOI:** 10.1186/s13104-018-3621-1

**Published:** 2018-07-31

**Authors:** Ioannis I. Spyroglou, Gunter Spöck, Alexandros G. Rigas, E. N. Paraskakis

**Affiliations:** 1 Department of Electrical and Computer Engineering, Democritus University of Thrace, 67100 Xanthi, Greece; 2 Department of Statistics, Alpen-Adria Universität, 9020 Klagenfurt, Austria; 3 Paediatric Respiratory Unit, Department of Paediatrics, Medical School, Democritus University of Thrace, 68100 Alexandroupolis, Greece

**Keywords:** Bayesian classifiers, Semi-Naive Bayes classifier, Asthma exacerbation, Prediction

## Abstract

**Objective:**

The achievement of the optimal control of the disease is of cardinal importance in asthma treatment. As the control of the disease is sustained the medication should be gradually reduced and then stopped. Nevertheless, the discontinuation of asthma medication may lead to loss of disease control and eventually to an exacerbation of the disease. The goal of this paper is to examine the performance of Bayesian network classifiers in predicting asthma exacerbation based on several patient’s parameters such as objective measurements and medical history data.

**Results:**

In this study several Bayesian network classifiers are presented and evaluated. It is shown that the proposed semi-naive network classifier with the use of Backward Sequential Elimination and Joining algorithm is able to predict if a patient will have an exacerbation of the disease after his last assessment with 93.84% accuracy and 90.9% sensitivity. In addition, the resulting structure and the conditional probability tables give a clear view of the probabilistic relationships between the used factors. This network may help the clinicians to identify the patients who are at high risk of having an exacerbation after stopping the medication and to confirm which factors are the most important.

**Electronic supplementary material:**

The online version of this article (10.1186/s13104-018-3621-1) contains supplementary material, which is available to authorized users.

## Introduction

Longitudinal studies are becoming increasingly popular in the field of medicine. Several artificial intelligence techniques have been developed for analysing this kind of data in several diseases [1, 2].

In addition numerous studies using exhaled volatile organic compounds, innovative exhaled inflammatory markers, telemonitoring data etc. have implemented a number of machine learning approaches to predict asthma exacerbation in children [3–6]. Bayesian network classifiers (BNCs) constitute a very important artificial intelligence technique [7]. The main advantage of BNCs compared to other classifiers (support vector machines (SVMs), logistic regression etc.) is that they are graphical models with the capability of displaying relationships between the predicting factors clearly. For that reason, BNCs seem to be a more appropriate classifier for studies of complex and multifactorial diseases such as asthma. In addition, BNCs with their graphical structure have the ability to show cause–effect relationships and therefore can be used to represent both direct and indirect causal relationships of the predicting factors of a disease [8].

Asthma is a complex chronic disease and the exacerbations of the disease usually occur after the discontinuation of medication [9]. Exacerbations are perceived by a progressive increase of asthma symptoms such as dyspnea, coughing, wheezing and by a decrease in spirometry measures such as forced expiratory volume in 1 s (FEV1) and peak expiratory flow (PEF).

The aim of this study is to predict and identify the patients that are at risk of having an asthma exacerbation after the medication cessation. The course of a patient after discontinuation of the medication is a very important issue. In some extreme cases an asthma exacerbation could lead even to patient’s death [10–12].

The identification of risk factors for asthma exacerbations remains a task not yet accomplished and BNCs can be an efficient method for detecting some of them.

## Main text

### Methods

A dataset of repeated measurements from 65 patients (195 observations, 2–4 measurements for each patient) aged from 1 to 14.5 years was gathered by the Paediatric Department of the University Hospital of Alexandroupolis, Greece during the period from 2008 to 2016. All of the patients have achieved good control of the disease and have interrupted their medication.

Additionally, it was necessary to include a time variable [ordinal categorical variable, i.e. the possible values ($t=1,2,\ldots$) are ordered ($1< 2 < \ldots$)] and a patient identity (id) variable (65 categories, one for each patient) in the BNC. A category change in a predictor variable through time may have different impact on different patients. The inclusion of id and time as variables deals with this matter as they will be contained in the conditional probability estimation of the class variable described in the next subsection. Prognostic factors used in the network are described in Table 1. The interval between the measurements is the medical surveillance interval of 6 months [13]. The first assessment (t = 1), is the one after discontinuation of the medication.

More information about the variables are given in the complete dataset provided in Additional file 1 [14–16].

**Table 1:** The encoding of the variables (nodes)

Categories	Prognostic factors
1: Yes 2: No	Food allergy, eczema, dyspnea, allergic rhinitis, daily symptoms, daily activities symptoms, breast feeding, smoking in prenatal period, bronchiolitis, exacerbation, pharmaceutical allergy, allergic conjunctivitis, pets, nocturnal symptoms
1: Female 2: Male	Gender
1: Intermittent 2: Persistent	Asthma category
0–10 (sum of the present allergens)	Allergens (categorization into 1 variable of all the following: *D. pteronyssinus*, *D. farinae*, olive, pellitory, graminaceae, pine, cypress, cat, dog, altenaria)
1: Toddler 2: Preschooler 3: Gradeschooler 4: Highschooler	Age
1: Hypoactivity (< 80%) 2: Normal activity (≥ 80%)	Forced vital capacity (FVC), FEV1
1: Non-significant (< 15) 2: Significant (≥ 15)	FEV1 reversibility
1: Poor asthma control (< 20) 2: Good asthma control ($20\le {ACT}\le {25})$ 3: Excellent asthma control (> 25)	Asthma control test (ACT)
Total points are 0–3 with more points indicating more control problems	Asthma Therapy Assessment Questionnaire (ATAQ)
1: Underweight 2: Normal 3: Overweight 4: Obese	Body mass index (BMI)

#### Bayesian network classifiers

BNCs are used for classifying instances into classes. Nodes represent the variables and arcs describe the probabilistic dependencies between them [17]. The combination of graph and probability theory, allows us to model complex relationships between a big number of factors. It is usual in BNCs the predictor variables to be called attributes and the dependent variable class variable. The goal of a BNC is to estimate the probability of each class of the class variable given the attributes based on the Bayes rule [18]:

1 $$\begin{aligned} P(C|A)=\frac{P(C)P(\mathbf {A}|C)}{P(\mathbf {A})}, \end{aligned}$$

where $\mathbf {A}=A_1,A_2,...,A_n$ and *n* the number of attributes. Also *P*(*C*) are the prior probabilities of the class variable *C* given by $P(c_i)=N_{i}/N$ ($N_{i}$ is the number of times category $c_i$ occurs in *N* samples). $P(\mathbf {A}|C)$ is the likelihood and $P(C|\mathbf {A})$ is the posterior probability. The algorithms used in this work are now described.

#### Naive Bayes classifier (NB)

NB is the most simple structure. It assumes that the attributes are conditionally independent given the class variable. In this case only the prior probability of the class and the conditional probabilities of each attribute given the class are required. So $P(C|\mathbf {A})$ is proportional to $P(C)\prod _iP(A_i|C)$ and taking the logarithm of the probabilities then a log-linear model is obtained somehow similar to a logistic regression model [18].

#### Tree—augmented Naive Bayes classifier (TAN)

It begins with the NB structure. Thereafter, a Hill-Climbing (HC) algorithm is used to find connections among nodes. The algorithm adds arcs until there is no further improvement in the performance of the classifier. An alternative is learning an one-dependence BNC with the use of Chow–Liu’s algorithm by maximizing certain scores (AIC, BIC, log-likelihood). In TAN the class variable has no parents and each one from the attributes has two parents at most, the class variable and another [19, 20].

#### Semi-Naive Bayes classifiers (SNBC)

Another alternative of BNCs is to transform the basic structure of a NB classifier onto a structure that takes into account dependencies between the attributes, while the tree structure is maintained. The basic idea of SNBC is to eliminate attributes in a way that the performance of the classifier is increased. There are two algorithms used. The filter forward sequential selection and joining (FSSJ) where the algorithm starts from a null BNC and adds attributes and the backward sequential elimination and joining (BSEJ) which starts with a full BNC and eliminates attributes in a way of increasing the performance [18].

### Results

The calculations were performed in R GUI 3.3.3 with the use of “bnclassify” and “bnlearn” packages [21, 22]. The last assessments of each patient are considered as test set. One major problem is that only 14.9% (29 out of 195) of the cases are high alert cases for an exacerbation. As a result there is a high risk that the classifiers will be biased towards the majority class. For this reason we decided to find an optimal cutoff different than the classic 0.5 to determine from which point and above a case will be considered as high alert. Therefore, a validation set which follows from repeated hold—out cross—validation in the training set is used to create a Receiver Operating Characteristics (ROC) curve to determine the optimal threshold with the minimum distance from the point (0,1) criterion [23]. A validation set must be used in order the results to be unbiased. The ROC curves are presented in Additional file 2.

The results of the implementations are tested by true positive (TP), true negative (TN), false positive (FP) and false negative (FN) values which give the following measures:

2 $$\begin{aligned} Sensitivity= & {} \frac{N_{TP}}{N_{TP}+N_{FN}},\end{aligned}$$

3 $$\begin{aligned} Specificity= & {} \frac{N_{TN}}{N_{TN}+N_{FP}},\end{aligned}$$

4 $$\begin{aligned} Accuracy= & {} \frac{N_{TP}+N_{TN}}{N_{TP}+N_{TN}+N_{FP}+N_{FN}}. \end{aligned}$$

The accuracy results are summarized in Table 2. The values inside the parentheses are the accuracy measures with the initial cutoff (0.5).

**Table 2:** Accuracy measures for BNCs

Bayesian network classifier	Accuracy	Sensitivity	Specificity
Naive Bayes	75.38% (89.2%)	72.72% (54.54%)	75.9% (96.2%)
TAN (BIC)	76.92% (86.15%)	72.72% (54.54%)	77.77% (92.6%)
TAN (AIC)	73.84% (84.61%)	81.81% (36.36%)	72.22% (94.44%)
TAN (log-likelihood)	75.38% (84.61%)	63.63% (27.27%)	77.77% (96.3%)
TAN (HC)	76.92% (86.15%)	72.72% (54.54%)	77.77% (92.6%)
FSSJ	53.84% (86.15%)	81.81% (36.36%)	48.15% (96.3%)
BSEJ	93.84% (89.2%)	90.9% (54.54%)	94.44% (96.3%)

**Fig. 1:**
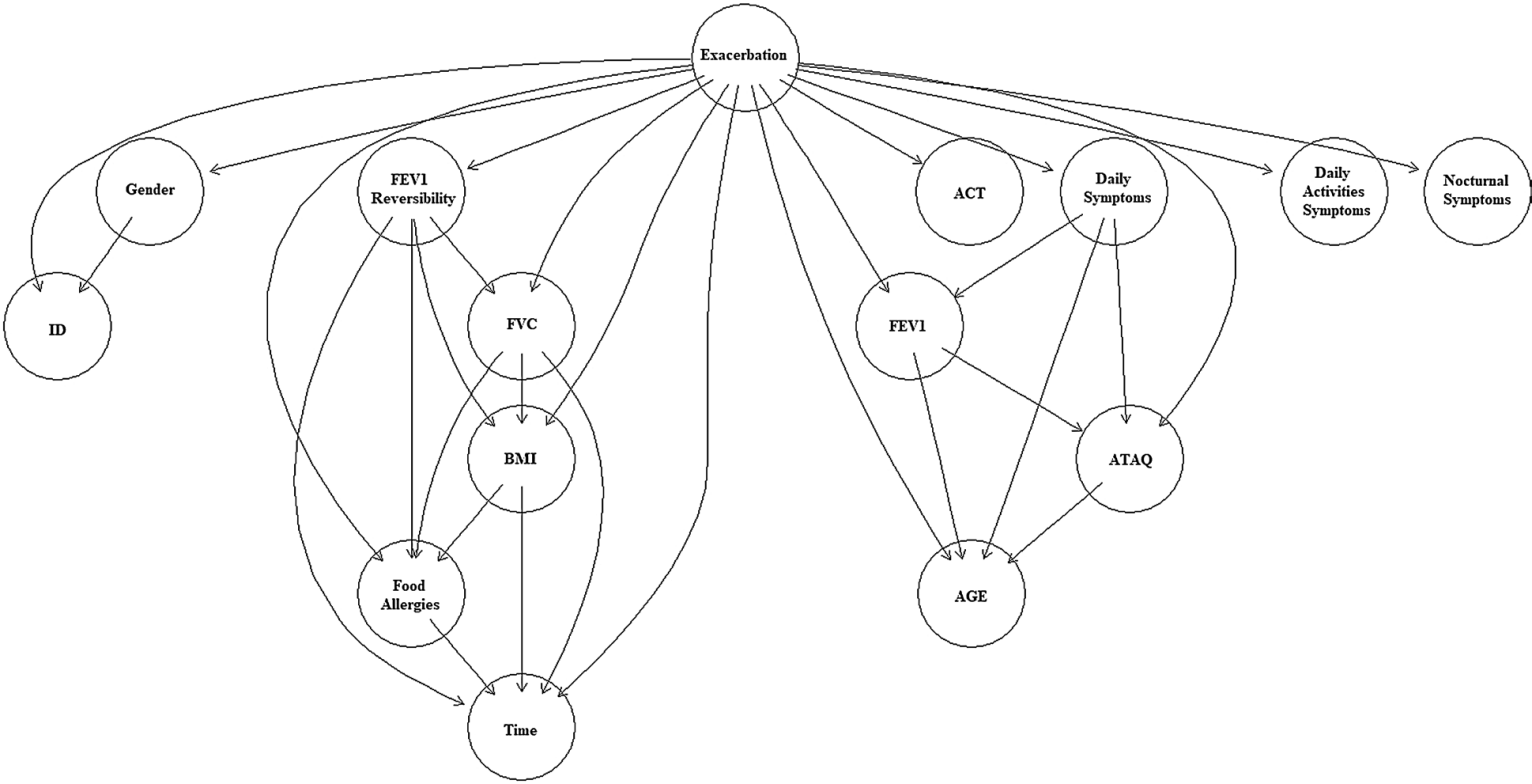
The structure of the BSEJ Bayesian classifier

The problem with this choice is that the sensitivity values are low and this is problematic in asthma exacerbation prediction. Therefore, it is required to change the normal cutoff to a lower value which is 0.06. As we can see in Table 2, the BSEJ algorithm results to a classifier that can identify high-alert cases better than the others. At the same time, the classifier has high specificity which leads to a more accurate model. The structure of the BSEJ classifier is presented in Fig. 1 showing how asthma exacerbation is affected by the attributes and the probabilistic relationships between them. These are described by the Conditional Probability Tables (CPT).

### Discussion

Our study showed that BNCs seem to be quite efficient in early prediction of high-alert asthma exacerbation cases. At this point, it is necessary to mention that multiple time points from the same patient may introduce bias in the final model, due to within-subject correlations. These correlations can be estimated through a GEE (Generalized Estimating Equations) logistic regression model [24]. In our case independence correlation structure seems to work well. However, in a larger scale (with more patients and time points) the classifier should be modified to deal with a potential more complex correlation. In addition, other classification techniques (SVMs, logistic regression) did not perform that well. Moreover, we have confirmed that gender, spirometric parameters, food allergies, age, day and night symptoms, ATAQ and ACT scores are the most important factors for a future exacerbation following treatment cessation. Using several algorithms we concluded that BSEJ algorithm has the best performance. The classifier derived by this algorithm contains 14 attributes. The advantage of this approach is that it takes into account the dependence that may exist between the attributes. Instead of using BSEJ we could have tried every possible combination of NB classifiers. The reason which led us to use BSEJ is that NB classifiers assume that the attributes are independent which is not valid in the case of asthma because the combination of some symptoms or patient’s characteristics could lead to an exacerbation. The importance of the factors can be examined through the CPTs which are provided in Additional file 3. For example, regarding BMI as has been shown in previous studies [25–28], the majority of the patients with low FVC% predicted who presented asthma exacerbation were obese. This shows the importance of those two factors combined, despite the fact that the effect of obesity on asthma exacerbations is still not very clear [29]. The presence of asthma symptoms during day, night or physical activities seems to favour an exacerbation as well. It is known that poor asthma control could lead to an exacerbation of the disease and all these can have significant effects in the quality of sleep [30]. Moreover, nocturnal asthma is associated with the increase of symptoms [31] and the need of additional medication. Additionally, the ACT score seems to play an important role in predicting future exacerbations [32], but we cannot rely only on this, because as the CPT of ACT shows, we have also a high percentage of Good Asthma Control in high-alert cases. Conclusively it seems that CPTs provide valuable information about important predicting factors the role of which in asthma prediction has been shown in numerous previous studies [27, 28].

Summarizing if we observe all the CPTs of the classifier, we will realize that all of the remaining factors seem to play an important role in asthma exacerbation prediction. This in turn indicates that asthma exacerbation prediction cannot depend only on few factors but it is a multi-factorial case. Most of the factors included are significantly associated with asthma exacerbations [10, 33]. In addition, a comparison with other studies using similar factors showed that the BSEJ BNC offered improvement in prediction accuracy. In [6] some of the factors included are the same as ours. Our BSEJ BNC seems to identify better high alert cases and at the same time exhibits higher overall accuracy in testing each patient’s last assessment. However, it would be very interesting to test how the BSEJ BNC will behave if environmental and socio-economic factors are also included [6].

### Conclusion

The goal of this study was to create a BNC using several factors for the prediction of high alert cases for an asthma exacerbation. The best performance was obtained with a classifier created with BSEJ algorithm. The fact that the prediction accuracy exceeds 90% (93.84%) with a sensitivity of 90.9%, shows that this classifier can be a useful tool for the clinical doctors. The basic advantage of using BNCs in asthma exacerbation prediction compared with the traditional clinical prediction methods which used simple parameters with low prognostic accuracy is that utilizes simultaneously a number of factors associated with exacerbation. Thus, a high accuracy in the exacerbation prediction is achieved.

## Limitations

The main limitation of this study is that the dataset is not large enough, so the statistical findings from this work should be studied in a larger scale in the future.

## Additional files

**Additional file 1.** The complete dataset used for the evaluation of BNCs in asthma exacerbation prediction.

**Additional file 2.** ROC curves of the BNCs with the use of validation dataset.

**Additional file 3.** The CPTs of the BSEJ Bayesian Classifier.

## Abbreviations

ACT: asthma control test
AIC: Akaike information criterion
ATAQ: Asthma Therapy Assessment Questionnaire
BIC: Bayesian information criterion
BMI: body mass index
BNC: Bayesian network classifier
BSEJ: backward sequential elimination and joining
FEV1: forced expiratory volume in 1 s
FSSJ: filter forward sequential selection and joining
FVC: forced vital capacity
GEE: generalized estimating equations
HC: hill climbing
LOGLIK: log-likelihood
NB: Naive Bayes
PEF: peak expiratory flow
ROC: receiver operating characteristics
SNBC: semi-Naive Bayes classifier
SVM: support vector machine
TAN: tree augmented Naive Bayes
