# Supplementary figures and images for: Evaluation of Bayesian classifiers in asthma exacerbation prediction after medication discontinuation
Source: BMC Res Notes. 2018 Jul 31;11:522. doi: 10.1186/s13104-018-3621-1 (PMC6069881; doi:10.1186/s13104-018-3621-1)

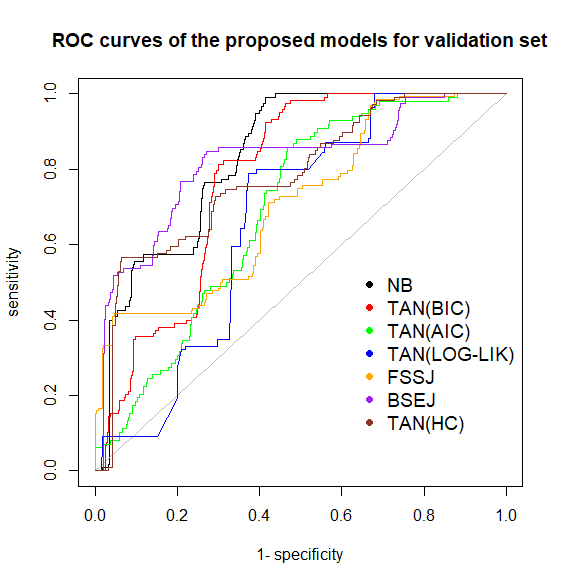

Supplement: Supplementary file 2 — Additional file 2. ROC curves of the BNCs with the use of validation dataset. [file 13104_2018_3621_MOESM2_ESM.tiff]
